# An Insight into Cellular and Molecular Mechanisms Underlying the Pathogenesis of Neurodegeneration in Alzheimer’s Disease

**DOI:** 10.3390/biomedicines11051398

**Published:** 2023-05-08

**Authors:** Yashumati Ratan, Aishwarya Rajput, Sushmita Maleysm, Aaushi Pareek, Vivek Jain, Ashutosh Pareek, Ranjeet Kaur, Gurjit Singh

**Affiliations:** 1 Department of Pharmacy, Banasthali Vidyapith, Banasthali 304022, Rajasthan, India; kailashaish@gmail.com (A.R.); aayushipareek26@gmail.com (A.P.); ashu83aadi@gmail.com (A.P.); 2 Department of Bioscience & Biotechnology, Banasthali Vidyapith, Banasthali 304022, Rajasthan, India; sushmitamaleys@gmail.com; 3 Department of Pharmaceutical Sciences, Mohan Lal Sukhadia University, Udaipur 313001, Rajasthan, India; vivek19j@gmail.com; 4 Adesh Institute of Dental Sciences and Research, Bathinda 151101, Punjab, India; drranjeetkaur93@gmail.com; 5 Department of Biomedical Engineering, University of Illinois Chicago, Chicago, IL 60607, USA

**Keywords:** Alzheimer’s disease, molecular mechanism, risk factors, amyloid plaques, tau tangles

## Abstract

Alzheimer’s disease (AD) is the most prominent neurodegenerative disorder in the aging population. It is characterized by cognitive decline, gradual neurodegeneration, and the development of amyloid-β (Aβ)-plaques and neurofibrillary tangles, which constitute hyperphosphorylated tau. The early stages of neurodegeneration in AD include the loss of neurons, followed by synaptic impairment. Since the discovery of AD, substantial factual research has surfaced that outlines the disease’s causes, molecular mechanisms, and prospective therapeutics, but a successful cure for the disease has not yet been discovered. This may be attributed to the complicated pathogenesis of AD, the absence of a well-defined molecular mechanism, and the constrained diagnostic resources and treatment options. To address the aforementioned challenges, extensive disease modeling is essential to fully comprehend the underlying mechanisms of AD, making it easier to design and develop effective treatment strategies. Emerging evidence over the past few decades supports the critical role of Aβ and tau in AD pathogenesis and the participation of glial cells in different molecular and cellular pathways. This review extensively discusses the current understanding concerning Aβ- and tau-associated molecular mechanisms and glial dysfunction in AD. Moreover, the critical risk factors associated with AD including genetics, aging, environmental variables, lifestyle habits, medical conditions, viral/bacterial infections, and psychiatric factors have been summarized. The present study will entice researchers to more thoroughly comprehend and explore the current status of the molecular mechanism of AD, which may assist in AD drug development in the forthcoming era.

## 1. Introduction

Neurological disorders are ailments that negatively impact the brain, spinal cord, and nerves all across the body [1]. They are generally marked by a slow, continual neuronal loss that eventually disrupts the stability of homeostasis in the nervous system of humans. As a result, processes such as abstract thought, locomotion, emotion, cognition, and memory are interrupted [2]. According to accessible statistics, these detrimental consequences could affect up to 2% of the world’s population [3]. One of the main categories of non-communicable diseases responsible for people’s degraded living standards is mental health and neurodegenerative disorders [4,5]. Globally, it is approximated that 44 million individuals suffer from AD or a corresponding form of dementia, while 8.5 million people have Parkinson’s disease (PD) [6,7]. For adults beyond 65, the risk of acquiring AD doubles every five years [8]. The predicted number of individuals in 2020 suffering from dementia was 55 million; the most prevalent reason for dementia was AD, contributing to 60–80% of total dementia cases [9]. By 2030 and 2050, this proportion is anticipated to nearly double in 20 years, reaching 78 and 139 million, respectively. Approximately more than 50 million individuals suffer from epilepsy on the planet. Every year, over six million people die from stroke, and the frequency of migraine has increased to more than 10% globally [10].

Neuronal communication is conducted through the synaptic junction formed by the intimate apposition of two cells. Signal transduction incorporates neurotransmitter production, which regulates the postsynaptic neuron activity, leading to their stimulation or inhibition [11]. The plasticity of synapses is highly crucial for memory and learning and involves both presynaptic and postsynaptic neurons. The key to neural survival and functionality is the synaptic integrity itself, which indulges the contribution of calcium signaling and intracellular protein coordination [12]. Failure in the optimal maintenance of adequate synaptic connections altered the neural stress levels due to excitotoxicity or abundant excitatory stimulation and neuronal apoptosis in extreme conditions, which are plausible conditions for the onset of neurological diseases [13]. Malfunctioning synaptic transmission and modulated post-synaptic receptor compositions are a few cellular modifications linked with abnormal neural functions [14,15].

The etiology of these disorders may be influenced by a number of risk variables including oxidative stress [16], genetic variations [17], endocrine disorders, age-related [18], inflammation [19], depression [20], hypertension [21], infection [22], diabetes [23], food supplements, exposure to chemicals, metabolic conditions, and vitamin deficiencies [24]. Furthermore, any structural, electrical, and biochemical alterations in the brain, spinal cord, and nerves lead to the occurrence of a specific number of symptoms comprising poor coordination, paralysis, seizure, muscle weakness, sensational loss, pain, confusion, and fluctuating levels of consciousness [25,26]. Neurological disorders are either associated with the central nervous system (CNS) or peripheral nervous system (PNS). The most frequently occurring neurological disorders involve stroke, Alzheimer’s disease (AD), amyotrophic lateral sclerosis (ALS), multiple sclerosis (MS), diabetic neuropathy, and PD [27].

NDs are increasingly acquiring a lot of medical emphasis due to the slow but steady increase in incidence. The leading causes of this progressive increase in age-related problems among all age groups are digital diabetes lifestyles, inactivity, hypertension, depression, hearing loss, midlife obesity, and smoking [28,29]. Preventive measures involving lifestyle modifications, physio or other forms of therapy, neurorehabilitation, pain management techniques, medications, surgeries undertaken by neurosurgeons, and a specified diet are examples of intervention strategies for neurological disorders [27,30]. The management of all of these disorders is challenging; drug distribution to the brain is constrained since the blood–brain barrier (BBB) prevents bulky molecules from passing through [31]. Therefore, it is crucial to devise a delivery mechanism that enables molecules to enter the damaged brain region without compromising the BBB system’s regular function [32].

## 2. Alzheimer’s Disease

The neurodegenerative disorder called Alzheimer’s disease mainly proceeds from brain cell degeneration, and ultimately leads to demise following dementia [33]. AD could be classified as familial or sporadic based on its occurrence. Familial AD comprises inherited genetic reasons associated with families, while sporadic AD emerges due to other unknown reasons excluding genetic ones [34]. AD has a protracted course and is featured by a gradual loss of memory and a general reduction in cognitive abilities. These devastating effects of neuronal degeneration reduce a patient’s competence to deal with everyday living tasks, frequently rendering their reliance on others for caregiving. Hundreds of millions of neurons are available in a healthy brain, which are specific cells that utilize electrochemical signals for dispatching information [35]. They carry impulses across distinct areas of the brain as well as from the brain to the body’s musculature and organs. This neural communication is disrupted by AD, which causes apoptosis and a decline in functionality [36].

A person with AD experiences distinct molecular and cellular modifications in the brain, which can be recognized using a microscope to look at the brain’s tissue after the death of an organism. Research is continually being undertaken to identify the abnormalities that may lead to AD and the actual reason for the consequence. Two distinct types of aggregates are the primary neuropathological indicators of AD [37]. The first is an extracellular amyloid (Aβ) peptide deposit (plaques). The second is hyperphosphorylated Tau protein fibrillary aggregation (tangles). It is typically proposed that neuroinflammation is another pathogenic characteristic of AD [38]. In exceptional instances, AD may be brought on by genetic flaws, but the preponderance of instances is sporadic and possesses no recognized etiology. AD has not been persistently connected to any environmental pollutants. Two proteins, tau and Aβ, appear to be irregularly processed and degraded, which tend to be directly associated with pathogenesis.

Normal aging allows the brain to shrink somewhat, yet oddly enough, a considerable proportion of neurons are not lost. Unfortunately, in the case of AD, many neurons stop functioning, break connectivity with the surrounding neurons, and ultimately die, causing significant harm. AD affects metabolism, repair mechanisms, and signaling, which are vital to neurons and their circuits. AD frequently begins by the destruction of neurons and their connections in the two brain sections linked to memory (i.e., the hippocampus and entorhinal cortex) [39]. Preceded by this, it affects regions of the cerebral cortex responsible for speech, thought, and societal interaction. Numerous other areas of the brain are subsequently destroyed. As time passes, an individual with AD progressively loses the ability to function independently and the ailment is inevitably lethal [40].

The symptoms of AD change with the phases of the disease. AD is categorized into three stages: preclinical or pre-symptomatic, mild, and dementia-stage, depending on the extent of cognitive deterioration. The primary and most-often appearing symptom is the transitory loss of memory with significantly prolonged memory loss, which can be evoked in most sufferers despite it not being present [41]. Short-term memory problems lead to difficulties with multiplexing and conceptual processing, which are followed by disorganization, problems with problem-solving, a lack of enthusiasm, executive performance, and reasoning. Decision-making dysfunction in the early phases could be mild to severe. Linguistic malfunction and visual-spatial performance degradation come next [42]. During the middle to late stages, it is common to experience nervous system-associated psychiatric indications such as lethargy, wandering, social disengagement, psychosis, and anxiety. Insomnia, olfactory malfunction, dyspraxia, and extrapyramidal motor indications including akathisia, dystonia, and Parkinson’s symptoms emerge late during the illness [43,44,45,46]. When age is considered, the disease does not seem to be gender specific.

Nonetheless, because of their longer lifespan expectancy, women comprise about two-thirds of the Alzheimer’s patient population [47]. AD proceeds at different frequencies. The average lifespan of any individual suffering from AD is approximately three to eleven years upon diagnosis, but some people survive for twenty years or longer. The duration of life may vary depending on the level of impaired functionality at the time of diagnosis. AD worsens more rapidly when vascular risk conditions such as hypertension are present [48].

## 3. Molecular Mechanism of Alzheimer’s Disease

### 3.1. Amyloid β Hypothesis

For over three decades, the amyloid hypothesis, proposed by G. Higgins, J. Hardi [49], and D. Selkoe [50], has been the dominant and most widely accepted mechanistic theory of how AD develops. According to their theory, the accumulation of oligomeric Aβ (oAβ)-peptides are responsible for the pathophysiology causing downstream events such as neuroinflammation, the formation of neurofibrillary tangles (NFT), and vascular injury, encouraging dementia and cognitive deficits [51]. Their original theory primarily focused on the frequent occurrence of AD in Down’s syndrome patients due to the generation of significant amounts of Aβ-peptides since the amyloid precursor protein (APP) gene is positioned on the three 21 chromosomes [52].

**Figure 1:**
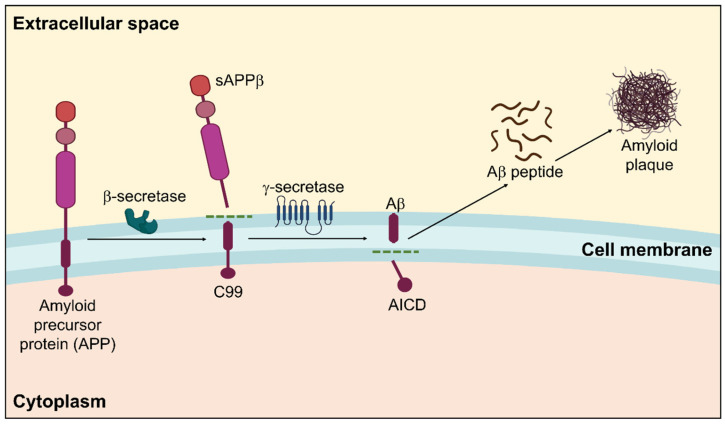
Amyloidogenic pathway in the pathogenesis of Alzheimer’s disease showing the formation of amyloid plaques. Within the membrane, β-secretase cleaves APP in the first instance, followed by γ-secretase. The extracellular amyloid-β that is released by the proteolytic breakdown of APP via the amyloidogenic pathway is susceptible to self-aggregation, resulting in the development of cytotoxic oligomers and insoluble Aβ fibrils/plaques.

Amyloidosis is a clinicopathological phenomenon where amyloid builds up in the body’s tissues and cells, generating amyloid plaques for various intricate reasons that eventually cause organ malfunction. It may run in the family or be acquired [53]. A systematic representation of amyloidosis in AD is displayed in Figure 1.

Amyloidosis is categorized into two types based on where amyloid fibers are deposited: localized amyloidosis, which affects a particular tissue in a specific place, and systemic amyloidosis, which affects the entire body [54,55]. These amyloid proteins make up amyloid plaques [56]. The primary element that significantly contributes to the pathophysiology of AD and is often regarded as the principal reason for AD development is the amyloid β peptide [53,57].

APP is produced by blood arteries, blood cells, neurons, and astrocytes in confined numbers and is a more significant precursor molecule than Aβ [58]. Multiple physiological functions for APP have been postulated thus far. APP is crucial for brain growth, memory, and neuroplasticity [59]. In addition to being able to safeguard neurons, it also controls intercellular relations, managing neuronal development and neuroplasticity [60].

Extracellular domains of the APP control cellular adhesion to support neural circuits. APP homodimers allow Aβ to activate calcium channels, which further modulate neural signaling and neurotransmitter discharge [61,62]. More precisely, K^+^-Cl^−^ cotransporter 2 (KCC2) is stabilized on cellular membranes due to direct protein–protein interactions between APP and KCC2, which can modulate hippocampal γ-aminobutyric acid inhibition (GABAergic inhibition). APP reduction causes KCC2 to degrade more quickly through ubiquitination and tyrosine phosphorylation, which impairs γ-aminobutyric acid type A (GABA_A_) receptor-regulated inhibition and GABA reversal potential depolarization [58]. Soluble amyloid precursor protein (sAPP) cleavage molecules including sAPPα and sAPPβ are responsible for several facets of APP functionality, wherein the role of sAPPα has been thoroughly described. sAPPα has been demonstrated to be preventative against Aβ-induced toxicity and serves a significant role in neuroplasticity/survival [60].

Moreover, the central nervous system’s early embryonic processes and neuronal stem cell growth can be mediated by sAPPα [63]. In response to specific neuroprotective agents, it has been proposed that sAPPα could suppress cyclin-dependent kinase 5 (CDK5) activation induced by excitotoxicity and take part in diverse excitoprotective mechanisms [64]. Notably, in APP-deficient mice, sAPPα expression alone is sufficient to reverse defects, indicating that sAPPα might facilitate most APP functioning. It has been revealed that APP mutations accelerate the production of Aβ, which results in senile plaques and peripheral neuron degenerative alterations [65].

Depending on their cleavage products, APP processing can be classified as either amyloidogenic or non-amyloidogenic. In APP processing, the major proteolytic enzymes are α-, β-, and γ- secretase. The principal β-secretase in the brain is beta-site APP-cleaving enzyme 1 (BACE1) and γ-secretase. The full-length APP is broken down by α-secretase, which releases the sAPPα ectodomain beyond the cellular membrane while leaving a C-terminal APP fraction of 83 amino acids inside the plasma membrane. This process is known as the non-amyloidogenic pathway [65]. The consecutive APP proteolytic cleavage via β- and γ-secretase complex constitutes the amyloidogenic pathway. When APP is broken down by γ-secretase, amyloid peptides with varying chain lengths such as Aβ-37/38/39/40/42/43 can be produced [66,67]. The two main Aβ species in the brain are Aβ42 and Aβ40. Aβ42 has a greater potency for aggregating due to the hydrophobicity of its two terminal residues, albeit soluble Aβ40 is significantly more abundant than Aβ42. Hence, Aβ42 is primarily responsible for constituting the majority of amyloid plaques that are neurotoxic [68]. Correspondingly, Aβ42 is considered a principal performer in commencing plaque building in the pathophysiology of AD [69]. Moreover, it has been established that AD may be distinguished from other dementias by employing the Aβ42/38 ratio and levels of Aβ38/42 in the cerebral spinal fluid (CSF) [70,71,72]. By boosting Aβ synthesis and decreasing the Aβ40/Aβ42 ratio, dysregulated APP function likely aids in the etiology of AD [73]. Aβ protein is a 40–42 amino acid short peptide of 4.2 kDa [65]. Misfolded proteins with a stable conformation are called amyloid proteins. Additionally, an abnormal build-up of Aβ causes neurotoxicity [53]. Monomeric Aβ segments are soluble molecules that coalesce to produce insoluble oligomers, which then develop into neurologic plaques. The transfer of Aβ by the receptor for density lipoprotein receptor-related protein-1 (LRP1) and the receptor for advanced glycation end products (RAGE) is among the strategies our body uses to remove Aβ from the brain [74,75,76]. Clinical research has demonstrated that an imbalance between Aβ production and elimination causes abnormal metabolism, which in turn, causes extracellular protein build-up and misfolding, resulting in the establishment of amyloid plaques [77,78].

Compared to other cell types, nerve cells generate more Aβ, which is crucial for intercellular signaling and other typical physiological processes of the CNS [79]. Individuals with traumatic brain injury and PD accumulate Aβ, indicating a link between amyloid and neurodegenerative disorders [80]. Chronic stress causes the body to respond by ramping up the production of neural proteins, which results in the build-up of by-products such as phosphate. The phosphorylation of APP can be facilitated by high phosphate concentrations in the protein production area. Additionally, β-secretase engages in the subsequent phosphorylation of APP processing, which causes Aβ deposition. However, there are several circumstances where the bodily function that regulates the concentration of Aβ can become uncontrolled. For instance, natural Aβ can stimulate the production of extra APP, which is then phosphorylated and processed to become amyloid, increasing the concentration of Aβ. In peripheral neurons, Aβ elevated concentrations might also stimulate the synthesis of APP and amyloidosis. In the brain, a portion of Aβ misfolds and accumulates, generating hydrophobic exogenous oligomers that acquire the shape of plaques and fibers that harm synapses and neurons [81]. Substantial evidence such as the existence of APP mutations in familial AD patients points toward Aβ as a principal factor in disease development.

**Figure 2:**
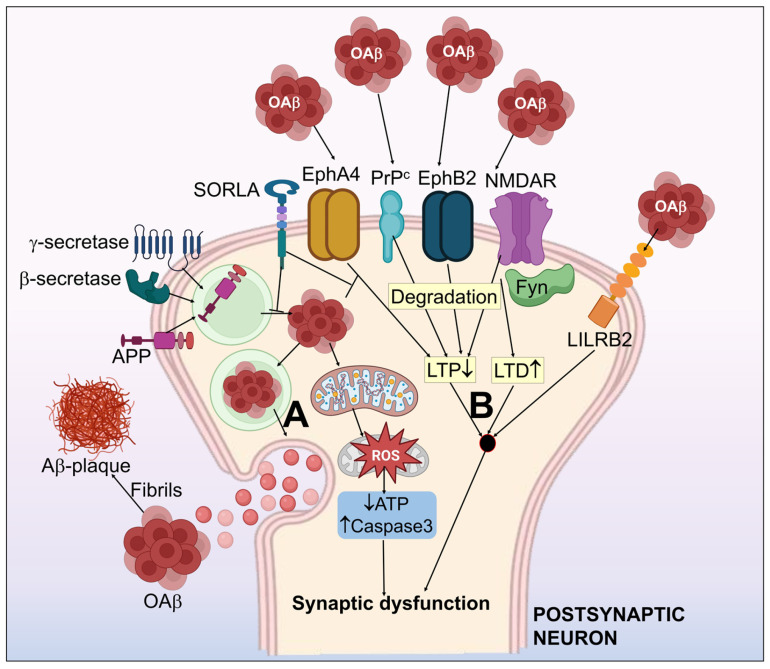
Systematic illustration of the amyloid-β hypothesis in Alzheimer’s disease. (**A**) APP processing to form Aβ, which simultaneously assembles as aggregates of the Aβ oligomer (oAβ) and form amyloid plaques. (**B**) Aβ-associated synaptic dysfunction by the impairment of LTP and LTD. Aβ receptors including NMDAR, PrPc, EphA4, EphB2 & LiLRB2 have been shown to induce synaptotoxicity by interaction with Aβ. EphA4-associated synaptic and cognitive malfunction may be inhibited by SORLA. Fyn kinase functions as an essential control mechanism for NMDAR related oAβ neurotoxicity. oAβ halts the normal mitochondrial function, which results in activated capsase-3, upregulated ROS, and decrease in ATP. This further worsens the synaptic dysfunction.

In the initial course of AD, patients have a synaptic malfunction; as the disease advances, synapses are lost. Synaptic loss is a primary pathogenic characteristic of AD and a reliable predictor of cognitive deterioration [82]. The degree of senile plaque development in the sick brain, however, does not always correlate with the severity of dementia that people with AD suffer. One viable argument is that soluble Aβ may indirectly contribute to AD pathophysiology by encouraging the development of neurofibrillary tangles [47]. The model for Aβ hypothesis in AD is displayed in Figure 2.

#### 3.1.1. oAβ Associated-Receptors

Even though the mechanisms behind oAβ-mediated synaptic malfunction have not yet been thoroughly defined, research findings have revealed a number of receptors that can induce Aβ synaptic toxicity. The receptors that are engaged with a reasonably high affinity toward Aβ comprise the leukocyte immunoglobulin-like receptor B2 (Lilrb2) [83], N-methyl-D-aspartate receptor (NMDAR) [84], cellular prion protein (PrPc) [85], ephrin type-B receptor 2 (EphB2) [86], and ephrin type-A receptor 4 (EphA4) [87].

##### LilrB2

The immune inhibitory receptor, LilrB2 is essential for maintaining the immune system’s homeostasis and repressing the immune system [88]. Current findings have correlated AD with LilrB2 and hypothesized that human LilrB2 is a potent oAβ receptor. In a mouse model of AD, paired immunoglobulin-like receptor (PirB) loss can also reverse cognition dysfunction. Cofilin and PirB engage mechanistically, and it appears that minimal amounts of the cofilin inactive phosphorylated form are present in the brains of individuals suffering from AD. The recruitment of cofilin-signaling modules due to the interaction between oAβ and PirB would cause actin depolymerization, culminating in synaptic malfunction and cognitive deficiencies [89]. A/LilrB2 inhibitors that may be bioactive such as ALI6 have been found to inhibit Aβ/LilrB2 interactions in vitro and reduce Aβ-induced neurotoxicity in the primary neurons [90].

##### NMDAR

In the neurological system, NMDARs, glutamate-initiated ion-gated cationic channels, are essential for exuberant synaptic transmission, development, and excitotoxicity [91,92]. Given that a subunit of NMDAR could coimmunoprecipitate with oAβ, they may interact directly [93]. Early phases of disease progress are probably when NMDAR activation by Aβ build-up takes place [94]. Aβ causes primary neurons to immediately influx Ca^2+^ by activating NMDARs that contain GluN2B, just like how NMDA stimulation occurs [95]. Furthermore, synthetic oAβ and Aβ derived from the AD brain could increase NMDAR-dependent long-term depression (LTD) [96,97]. These modifications could result from boosted NMDAR endocytosis and decreased NMDAR expression brought on by Aβ [98]. These findings demonstrate that the partial inhibition of NMDAR jaded stimulation with antagonists of NMDAR restores Aβ-initiated long-term potentiation (LTP) deficits and cognitive performance in distinct animal models, suggesting the importance of NMDAR in AD [99].

##### PrPC

PrPC is a relatively conserved protein in vertebrates at all developmental periods [100]. PrPC sized in the neuron’s pre/post-synaptic divisions, and expressed in different brain regions, specifically the hippocampus and cortex [101,102]. Numerous processes such as neuronal differentiation, neurite outgrowth, and survival could be regulated by PrPC [103]. PrPC was discovered to be a potentially high-affinity receptor for oAβ by a genome-wide association study (GWAS) [104]. Later research established that oAβ selectively binds to a 95-111aa N-terminal stretch of PrPC, particularly in oligomers of higher molecular weights [105]. In various AD mice models including APP/presenilin-1 (PS1), PrPC ablation substantially corrected synaptic LTP impairments by oAβ [106]. On the other hand, oAβ responses with PrPC had little or no impact on the formation of Aβ plaques and glial activation [107,108].

##### EphB2

In the maturation and adequate development of the nervous system, the Eph family that belongs to receptor tyrosine kinases perform crucial functions [109,110]. B-class ephrin ligands and Eph receptors coordinate bidirectional signaling, stimulating signals in both the receptors and ligand-bearing cells. Eph receptors govern neuroplasticity, dendritic spine formation, and neuronal-glial transmission in the brain [111]. It is intriguing that several neurological diseases including AD have recently been linked to Eph receptors and their function in synapse formation [112]. Hippocampal neurons exposed to oAβ exhibit lowered amounts of membrane EphB2, possibly due to cross-regulatory engagement between NMDAR and EphB2. EphB2′s fibronectin repeat section is where oAβ binds, leading EphB2 to be endocytosed and decomposed [113]. Surprisingly, LTP and cognitive memory deficits were corrected in an AD mice model by overexpressing EphB2 in the dentate gyrus [86]. Additionally, EphB2 overexpression can reverse the α-amino-3-hydroxy-5-methyl-4-isoxazolepropionic acid receptor (AMPAR) and the NMDAR level decline is brought on by oAβ [114].

##### EphA4

When it comes to synaptic function, EphA and EphB serve opposite functions. During synaptic trimming, EphA4 physiological activation via an astrocytic ephrinA3 ligand at postsynaptic densities causes dendritic spine retraction via CDK5 and ephexin1. As a result, in the mouse brain, the deletion of EphA4 leads to more spines than in the wild type, which is also longer and less arranged. Interestingly, research within the last few years has correlated EphA4 to AD [115]. EphA4 messenger RNA (mRNA) expression has been observed to rise in synaptosomes in AD patients [116]. The human hippocampus also shows EphA4 accumulation in the areas around senile plaques and the AD brain exhibited higher levels of active EphA4 [117]. Since EphA4 in the hippocampus neurons are inhibited or not present, oAβ could cause the activation of EphA4 by binding to it, eliminating synaptic loss [118,119].

### 3.2. Tau Pathology toward Neurofibrillary Tangles

**Figure 3:**
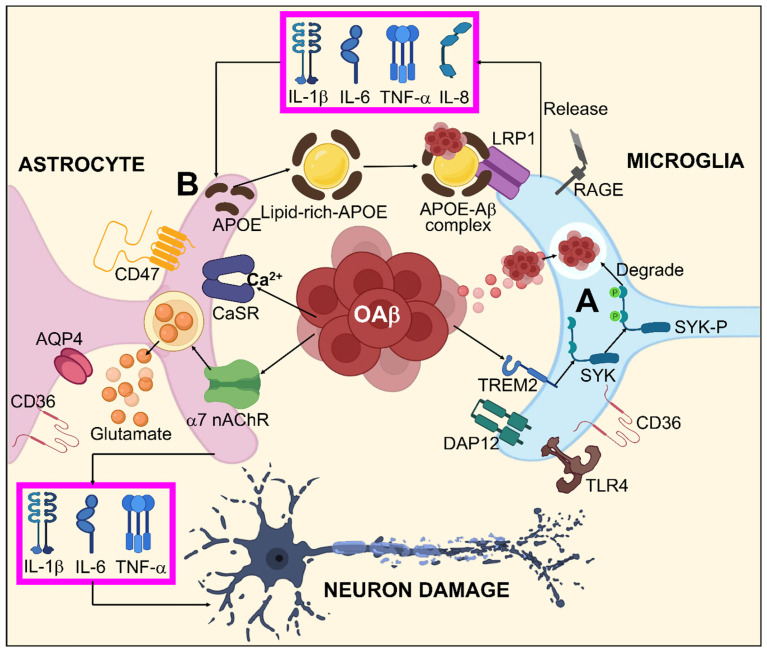
Systematic illustration for Aβ-mediated glial response in AD. (**A**) oAβ might activate microglia by binding to different Aβ receptors including TLR4, RAGE, LRP1, CD36, and specifically to TREM2, which stimulates the SYK pathway via DAP12 inducing Aβ degeneration. (**B**) Aβ dependent astrocyte dysfunction by enhanced interactions between Aβ/APOPE and LRP1 results in astrocyte activation by releasing TNF-α, IL-1β, IL-6, and IL-8. Furthermore, oAβ is also capable of direct astrocyte activation by AQP4, CD36, α7-nAchR, CD47, and CaSR. This astrocyte activation leads to neuronal damage through TNF-α, IL-1β, IL-6, and excitotoxication/irregulated homeostasis of glutamate.

Tau is a cytosol protein mostly available in axons and is a neuronal microtubule-associated protein. The microtubule-associated protein tau (MAPT) gene possessing 16 exons is localized on chromosome 17, which encodes human tau [120,121,122]. Tau helps microtubules and related proteins assemble and remain stable [123]. By engaging on microtubules using its extensively conserved microtubule-binding repeat domains, tau also aids in regulating microtubule processes such as axon transport and neurite growth [124,125]. Microtubules oscillate between a stable phase and dynamical instability; efficient neural transmission and survival depend on optimal balancing among these two states [126]. This system relies on tau phosphorylation, which reduces tau’s capability for microtubules while maintaining their dynamic character to support the optimal neuron activity [127,128]. However, aberrant or excessive Tau phosphorylation reduces the integrity of microtubules, resulting in an elevation in neurite branching, a deduction in axonal transit, and synapse retraction, as shown in Figure 3 [126,129]. Neurodegenerative conditions like AD, ALS, and PD are featured by hyped phosphorylation of tau and the consequent micro tubular instability [130,131]. In the AD brain, hyperphosphorylated Tau could develop into oligomers, filaments of paired helical, and eventually neurofibrillary tangles [125,132,133,134]. Tau is more challenging for phosphatases to dephosphorylate once it has aggregated [135]. Oligomeric Tau could take effect as a “seed” and encourage additional Tau proteases in neighboring neurons to condense into fibrils [134,136,137]. It has been discovered that tau oligomers are the primary cause of axonal transport deficiencies in neurons, which can result in neural death [137,138]. Immunohistochemical staining was developed by researchers E. Braak and H. Braak to stage neuro-pathological Tau aggregation in the brain, and it has since been improved to make it easier for pathologists to determine the level of Tau deposition and whether AD needs to be identified in a post-mortem of the patient [139,140].

The presence of Aβ, neural inflammation, enzymes, and oxidative stress that modulate phosphatases and kinases can all impact the phosphorylated tau-protein conformation [141,142]. Microtubule affinity-regulating kinase (MARK), CDK5, and glycogen synthase kinase 3 (GSK3) are the three enzymes that likely have a major impact [143,144,145]. The formation of Tau into neurofibrillary tangles is in close alliance with the neurodegeneration (i.e., neural demise) and brain atrophy seen in AD [139,146,147]. The hippocampus and entorhinal cortex are the first areas of the AD brain to be impacted, accompanied by areas of the temporal lobe and neocortex. During this period, patients may experience moderate cognitive impairment (MCI) [148]. The degeneration then progresses to the frontal portions of the cortex and occipital lobe, resulting in delayed personality alterations and trouble accomplishing daily tasks [149,150]. These frontal portions shrink while the ventricles are expanded. The primary pathogenic driver of ventricular enlargement and cortical atrophy is believed to be neuronal loss [151].

### 3.3. Mitochondrial Dysfunction and Reactive Oxygen Species (ROS) Generation

According to multiple evidence-based studies, the pathophysiology of AD may be influenced by mitochondrial dysfunction [152]. As a result of Aβ aggregation in the mitochondria of AD brains, disrupted mitochondrial conformation, reduced adenosine triphosphate (ATP) release and respiratory function, increased mitochondria-mediated oxidative stress, and poor mitochondria dynamics occur. The brain mitochondria of the patients suffering from AD and mouse models have both been reported to possess Aβ, which is responsible for neurodegeneration [153]. Irregularities in mitochondrial structure and functioning are associated with elevated mitochondrial Aβ. For example, reduced energy consumption related to mitochondria was noted in brain areas connected to amyloid plaques. Aβ also causes anomalies in mitochondrial function; due to decreased energy generation, abnormal alterations are also observed in the mitochondrial dynamics. Additionally, proteins linked to enhanced mitochondria fission and reduced fusion of mitochondria are amplified by Aβ exposure [154]. Unfortunately, it is still uncertain how mitochondrial dysfunction contributes to AD.

**Figure 4:**
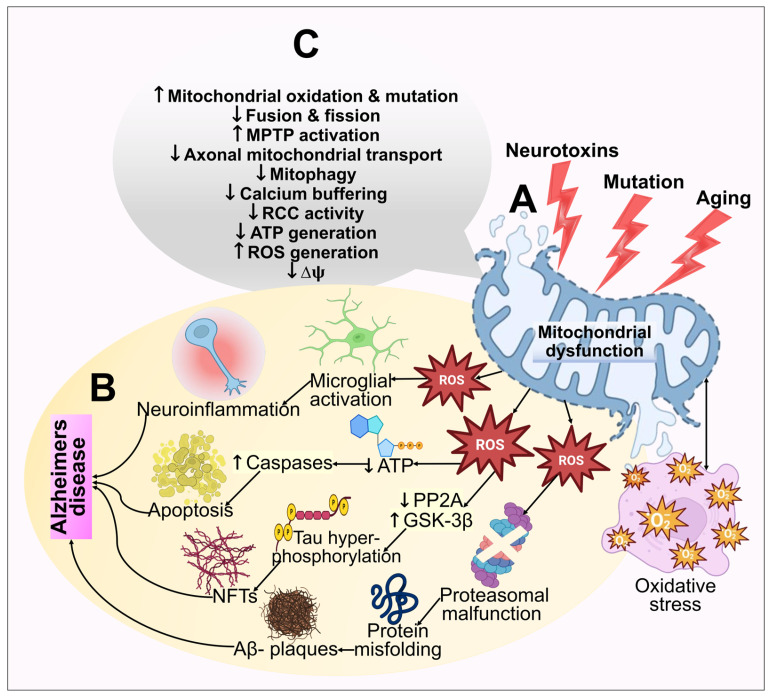
An illustration of the mitochondrial dysfunction and oxidative stress in the pathogenesis of AD. (**A**) Multiple age-related processes, mutations, and toxic fluctuations such as metal exposure can all adversely affect mitochondria. Mitochondrial dysfunction further results in bioenergetic deficits, calcium imbalance, and free radical production. This causes oxidative stress, which exacerbates mitochondrial impairment, synaptic malfunction, cognitive decline, and memory loss. (**B**) The cellular redox equilibrium is disrupted by ROS generation or a compromised antioxidant arrangement, which leads to an oxidative imbalance and excessive ROS output. By adversely influencing mitochondrial energy reserves, disrupting energy metabolic processes, and impairing dynamics and mitophagy, elevated ROS reduces mitochondrial ΔΨm and ATP production. Caspase activity also rises as a result of ROS, which additionally starts the apoptotic process. However, excessive ROS generation inhibits phosphatase 2A (PP2A), which leads to glycogen synthase kinase 3 (GSK3) activation. This results in tau hyperphosphorylation and NFT buildup. (**C**) The functions of the mitochondria that are extensively hampered in AD have been highlighted.

The oxygen consumption and metabolic rate of neurons are exceedingly high. As a result, to generate energy by oxidative phosphorylation, neurons depend on the numerous mitochondria in brain regions. ROS are primarily generated in mitochondria as by-products of oxidative phosphorylation, and routine homeostatic action in mitochondria frequently blocks excessive ROS formation. Furthermore, there are indications that oxidative assaults are critical in AD pathophysiology [155]. The idea that oxidative stress might be what causes AD pathogenesis triggered by Aβ is supported by the finding that oxidative stress emerges earlier in AD [156]. Aβ-peptides can elicit mitochondrial ROS generation, which releases cytochrome c and an apoptosis-inducing factor, causing malfunction of mitochondria, apoptosis, and the death of neurons [154,157]. In AD, appoptosin overexpression can cause the intrinsic caspase pathway to be activated. Prominently, decreased appoptosin expression can guard against Aβ’s neurotoxic effects [158]. Amyloid-binding cyclophilin D alcohol dehydrogenase are the few other mitochondrial proteins that have depicted a role toward mitochondrial dysfunction [159,160,161]. Mitochondrial dysfunction and oxidative stress in the pathogenesis of AD are illustrated in Figure 4.

### 3.4. Nitrosative Stress

Nitrosative stress arises when various defensive mechanisms fail to balance the formation of reactive nitrogen species (RNS), which harms intracellular constituents. The main component of RNS is nitric oxide (NO), which acts as a signaling molecule to control synaptic plasticity, neurotransmission, and brain growth. Significant cognitive impairment aligned to synapse malfunction and glial activation has been linked to nitrosative stress [162,163]. Due to S-nitrosylation, nitric oxide released as a consequence of Aβ in AD has been identified to trigger fission in mitochondria, resulting in synaptic dysfunction and neuronal death [164]. Since higher S-nitrosothiol (SNO)-CDK5 amounts were found in post-mortem samples of the AD brain and not in healthy samples, it has been determined that enhanced SNO-CDK5 activity possesses a part in the progression of AD [165]. Assessing the role of S-nitrosylation in nitrosative stress-initiated AD pathogenesis is made more accessible by the massive neuronal atrophy in the AD brain, accompanied by elevated S-nitrosylation of the peptides and a considerable proportion of altered sites of cysteine [166].

### 3.5. Protein Oxidation and Lipid Peroxidation

Multiple evidence-based findings imply that ROS might be crucial in the emergence of neurodegeneration in AD. ROS and RNS build up over time, which results in protein oxidation and lipid peroxidation. The brain also possesses an elevated proportion of unsaturated lipids, a high metal ion concentration, an elevated oxygen usage rate, and a poor antioxidant system. Consequently, both protein and lipid oxidation are particularly dangerous for the brain.

#### 3.5.1. Lipid Peroxidation

The oxidative breakdown of lipid molecules is alluded to as lipid peroxidation. Removing the H-atom from lipids in the cellular membrane by free radical species sets off a series of events that lead to cellular membrane destruction. Due to its elevated oxygen uptake, a large quantity of redox metal ions, weakened antioxidant defense system, and a higher proportion of polyunsaturated fatty acids (PUFAs), the brain is thought to be particularly sensitive to lipid peroxidation [167]. By Michael’s adduction to the amino acids histidine, lysine, and cysteine, the by-product 4-hydroxy-2-nonenal (HNE) might establish covalent connections with proteins. The experimental studies conducted by Tamagno et al. [168] showed that elevated lipid peroxidation triggers the activation of BACE 1, which in turn elevates the production of Aβ. According to a study, the AD hippocampus contains higher levels of HNE-histidine Michael adducts, and the covalent alteration of the histidine side chain of Aβ leads to greater tau protein aggregation [169]. Notably, c-Jun N-terminal kinase (JNK) pathways can be activated by lipid peroxidation and Aβ (production in neurons), which results in programmed neuronal cell death. HNE is a very deadly substance that prevents muscarinic cholinergic and metabotropic glutamate receptors from coupling to phospholipase C-coupled GTP-linked proteins [170] and is an aldehydic derivative of lipid peroxidation. HNE also halts glutamate transport in cortical astrocytes and glucose transport in hippocampal neuronal cells [171].

#### 3.5.2. Protein Oxidation

Protein carbonyls and 3-nitrotyrosine (3-NT) concentrations are elevated in response to cell protein oxidation. The interaction of the superoxide anion can produce protein carbonyls, which fragment the protein’s backbone. Moreover, the specified ROS attack on the side chains of several amino acids including lysine, proline, and arginine might lead to protein carbonyl generation. Various investigations have revealed the creation of Michael adducts amongst cysteine, lysine, and histidine and residues, which results in advance glycation end products (AGEs) [172,173]. According to Bota et al. [174], a variety of human disorders may be caused by the inadequate elimination of transformed proteins and deposits. There seems to be strong evidence that the protein carbonyl level was enhanced in the parietal lobule and hippocampus of individuals suffering from AD by 37% and 42%, respectively [175]. Currently, utilizing a redox proteomic strategy, researchers have uncovered additional altered proteins in distinct portions of the brain in AD patients including peptidyl-prolyl-cis, trans isomerase 1 (Pin1), enolase, creatine kinase BB, ubiquitin carboxyl-terminal hydrolase L-1 (UCHL-1), heat-shock 71, and dihydropyrimidinase-related protein 2. Furthermore, ATP depletion in brain cells can result in aberrant tau protein phosphorylation, possibly contributing to the start of AD [176].

### 3.6. DNA Damage

DNA is nucleic acid found in the mitochondria (mtDNA) and nuclear (nDNA) material of living cells. Multiple lines of research have indicated that ROS generated as an oxidative phosphorylation by-product and environmental subjection to chemicals and radiation target nuclear and mitochondrial DNA for the genotoxic attack. Furthermore, investigations have demonstrated that because mtDNA is situated near the oxidative phosphorylation cascade, it is more vulnerable to genotoxic attack than nDNA [177].

The Tau protein, in conjunction with its function in microtubule dynamics, is essential for protecting the genomic DNA of neurons from oxidative stress and ROS. The modification in the tau protein may impair nucleic acid protection mechanisms and make hippocampus neurons more vulnerable to ROS-induced oxidative stress to their nuclear RNA and genomic DNA in AD patients. It has been established that ROS can damage DNA strands and play a role in subsequent AD disease-causing processes [178]. Mullaart et al. [179] found a two-fold rise in DNA destruction in neurons of the AD brain. They theorized that this might be one of the early identifiable pathogenic events in the transition from the normal to the AD brain. 8-Hydroxyguanine (8-OHG) is the most protruding DNA marker in most biological samples including blood cells, urine, and brain tissues [180]. 8-Oxoguanine-DNA glycosylase (OGG1) is a bifunctional enzyme that has apurinic/apyrimidinic lyase and DNA glycosylase properties. Evidence from research has shown two single-nucleotide polymorphisms in OGG1 caused by the substituted amino acid A53T and A288V. These polymorphic OGG1 proteins with the A53T and A288V mutations were found in 2007 in late-stage brain tissue AD patients but not in the controls [181].

### 3.7. Glial Cells in AD

Another characteristic of AD is neuroinflammation, which appears as gliosis and is marked by the activation and proliferation of the two main glial cell types in the brain, astrocytes and microglia. Numerous recently discovered AD risk genes such as triggering receptors expressed on myeloid cells-2 (TREM2) are only expressed in glial cells or are greatly concentrated in them. As a result, current research has placed a lot of emphasis on the probable impact that the glia may serve in the pathogenesis of AD. Pathogenic tau and Aβ species can bring neuroinflammation and gliosis. Glial cells and inflammation can control the development of Aβ and tau in a reciprocal manner. In general, it is thought that inappropriate microglial and astrocyte activation is a harmful event during the initiation of AD, and that blocking the formation of pro-inflammatory cytokines and the malignant glial responses to pathogenic Aβ and tau may prevent AD pathogenesis.

#### 3.7.1. Aβ Pathogenesis and Glial Cells

In AD, inappropriate Aβ collection seems to be what starts the inflammatory processes. In such a situation, oAβ might increase proliferation by activating microglia. Microglia are indigenous immune cells that govern homeostasis in the brain by managing immunological functionality, phagocytosis, and plays a reparative role [182]. Since active microglia vigorously phagocytose and destroy oAβ, they may be preventive in the beginning stages of AD. Furthermore, by the secreting brain and glial-derived neurotrophic factors, microglial activation may aid neuronal healing [183].

Moreover, active microglia can also increase oxidative stress and release pro-inflammatory cytokines such as IL-1β and IL-6 in AD and tumor necrosis factor-α (TNFα) [184,185]. Conversely, overactive microglia may mitigate synaptic function by promoting phagocytic synaptic trimming. Hence, due to putative adverse effects related to inflammation, neurotoxicity, and degeneration, chronic microglial excitation during AD development may be harmful. Neuroinflammation can also worsen Aβ build-up by interfering with phagocytic Aβ intake and elimination. The ability of tert-butyl hydroperoxide, lipopolysaccharide (LPS), IL-1β, and prostaglandin E2 to decrease the phagocytosis of microglia and increase Aβ accumulation has been demonstrated [186].

In the general brain form of glial cells, astrocytes define the boundaries dividing the nerve and non-nerve tissue throughout the meninges and vascular areas. Functional barriers created by astrocyte boundaries and scars restrict the movement of inflammatory cells into the CNS parenchyma. Astrocytes are therefore vital to limiting inflammation in the CNS [187]. Astrogliosis occurs as an outcome of Aβ accumulation and proinflammatory cytokines generated by activated microglia during the pathophysiology of AD. Active astrocytes possess a bidirectional impact on AD. On one hand, they can encourage the breakdown and removal of Aβ primarily via the production of apolipoprotein E (APOE), an essential modulator for Aβ clearance [188,189,190]. On the other hand, by releasing RNS, ROS, and pro-inflammatory cytokines that obstruct synaptic development and the growth of axons, activated astrocytes might worsen inflammation [191,192,193]. Current investigations have defined a particular subtype of A1 astrocytes that is reactive activated by TNF, IL-1, complement component 1q (C1q), and mitochondrial fragment out bursting from active microglia, suggesting that microglia might serve a substantial part in maintaining astrocytic activation [194]. A1 astrocytes are more prevalent in neurodegenerative conditions like AD where they have been demonstrated to have diminished neuroprotective functions such as supporting neurogenesis, outgrowth, and synapse formation as well as incapacitated phagocytic capabilities. A1 astrocytes can also cause neurons and oligodendrocytes to die [195]. Even though the exact mechanisms underlying the interactions between Aβ and glia are still vague, mounting evidence suggests that several glial receptors including Fc γ receptors IIb (FcγRIIb) [196], LRP1, cluster of differentiation 36 (CD36) [197,198], complement receptor 3 (CR3) [199], RAGE [200], Toll-like receptor 2/4 (TLR2/4) [201], and TREM2 perform pivotal roles in facilitating Aβ-mediated glial responses and functionalities. Aβ associated glial responses in AD are illustrated in Figure 3.

#### 3.7.2. Tau Pathogenesis and Glia

Considering that several tau transgenic animal models and tauopathy sufferers exhibit gliosis even in the absence of Aβ pathology, pathogenic tau species can also activate microglia and astrocytes. Pro-inflammatory cytokines like IL-1β, IL-6, and TNF-α can be secreted more readily as a result of tau-mediated microglial activation [202,203,204]. Recent transcriptome studies have shown the involvement of nuclear factor kappa B (NF-κB) and nucleotide-binding domain, leucine-rich–containing family, and pyrin domain-containing-3 (NLRP3) in the process of tau-activating microglia to cause inflammation, despite this, the exact mechanism is still unclear [205,206]. Aβ and tau appear to retain similar pathways for activating microglia because Aβ also activates NF-κB signaling and the NLRP3-ASC inflammasome [207,208]. Through assimilating and destroying pathogenic tau from the AD brain, wherein the related mechanisms of the process remain obscure, microglia can drastically improve tau elimination [209]. Countless studies have revealed that the CX3C motif chemokine receptor 1 (CX3CR1) is essential for promoting tau breakdown and microglial phagocytosis. CX3CR1 interacts with tau and hyperphosphorylated tau to a limited extent [210]. Lack of CX3CR1 affects tau uptake by microglia in vitro and encourages hyperphosphorylated tau aggregation in vivo [211]. Microglia may have an impact on tau propagation through exosome generation in addition to influencing tau elimination, as exosome propagation can be stopped by depleting microglia or inhibiting exosome synthesis [212,213,214]. As a result, pathogenic tau and microglia activation may generate cyclical pathogenic episodes throughout the development of AD.

**Figure 5:**
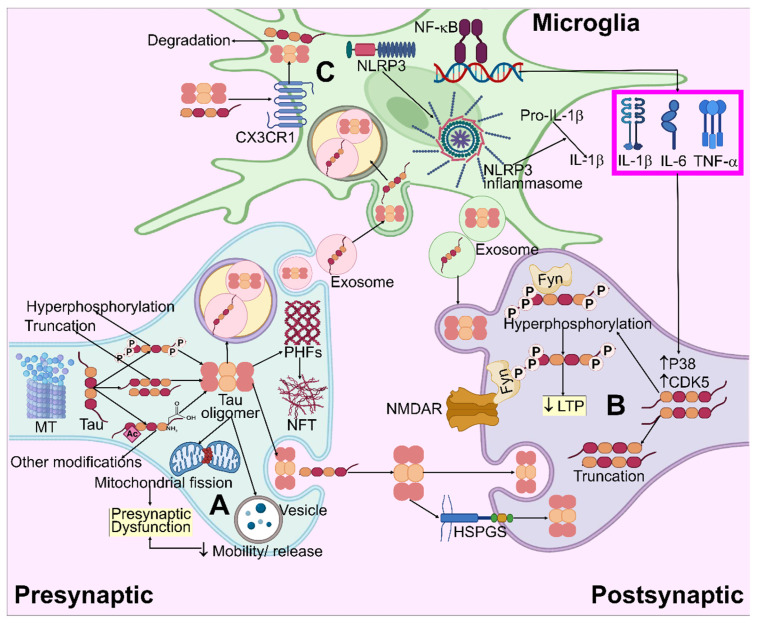
Systematic illustration for tau pathogenesis in AD. (**A**) Tau aggregation and oligomer formation result in mitochondria fragmentation and the impairment of vesicle mobility/release, which causes presynaptic dysfunction. (**B**) Truncated and hyperphosphorylated tau species enter the post-synapse and modulate NMDAR/Fyn complexes, leading to LTP impairment. By means of the heparan sulfate proteoglycans (HSPGs)-mediated route, extracellular pathogenic tau species may be embodied in neurons, causing intracellular tau accumulation. (**C**) Extracellular tau interacts with CX3CR1, enters the microglia, and degrades. Microglial NF-κB and NLRP3 inflammasome pathways are activated by tau, which allows for the release of pro-inflammatory cytokines. These cytokines enhance the CDK5 and P38 activity, which leads to increased hyperphosphorylation.

Astrocytes directly influence the pathophysiology of tau. Tau accumulation can be seen in the astrocyte nucleus in the AD brain, despite tau primarily building up in the neurons [215,216]. Tau aggregation in astrocytes affects astrocyte functioning, causes neurodegeneration, and encourages apoptosis through a series of degenerative processes including accelerating the collapse of BBB and triggering the expression of low-molecular-weight heat shock proteins [217,218,219,220]. Pathogenic tau may also impair astrocytic-mediated glutamate transport, leading to glutamate deposition in the brain and subsequent excitotoxicity [221,222]. The model for the tau hypothesis and its impact on microglia in AD is illustrated in Figure 5.

To sum up, it seems likely that Aβ initiates tau pathogenesis in AD, where pathogenic tau and Aβ jointly cause gliosis and neuroinflammation. Inflammatory elements and reactive glial cells work synergistically to accelerate the development of Aβ and tau pathophysiology to worsen the neurodegeneration.

### 3.8. Proteasomal Dysfunction

By eliminating proteins that are inappropriately folded or clumped together, the ubiquitin-proteasome pathway (UPP) helps to maintain cellular integrity. When this system for removing undesirable protein complexes is disrupted, toxic and improperly folded proteins gather in brain cells, which is thought to be a pathogenic characteristic of AD. This route is crucial for the efficient elimination of aberrant protein garbage, which is essential for the viability and stability of neurons [223]. In order to continually remove defective proteins from neurons and block the aggregation of inappropriate proteins, the optimal functioning of UPP is of utmost importance [224].

Recent research has exhibited that the intracellular deposit of phosphorylated tau and Aβ protein clumps in AD patients directly impairs UPP. Brain tissue from patients with early AD had much less proteosome activity. The proteasome activity is reduced by 56% in AD patients due to the intraneural accumulation of paired helical filaments, which inhibits the proteasome. As a result, in the AD brain, the failure of UPP to remove phosphorylated tau and paired helical filament ultimately causes neuronal death [225]. Current findings have shown a strong correlation between ubiquitinated synaptic tau and hyperphosphorylation. This stable oligomerization of ubiquitinated synaptic tau results in elevated proteasome elements, proposing that a failure of the ubiquitin-proteasome system causes AD [226,227]. Although extant research has asserted a strong correlation between the build-up of hyperphosphorylated tau and malfunctioning UPP, none of these studies has explicitly stated whether the hyperphosphorylated tau is to blame for the UPP machinery’s impairment or the other way around [228]. As a result, further research employing cellular models are required to pinpoint the pathogenic event that causes aberrant neuron activity in AD patients.

### 3.9. Neuroinflammation

Infection, trauma, or toxic materials can cause a complicated series of inflammatory reactions in the brain system known as neuroinflammation. Microglia and astrocytes are important cells that indulge in inflammatory processes in the CNS and neuronal cells. By showing the existence of reactive microglia in the substantia nigra portion of post-mortem brain tissue from PD patients, Mc Geer et al. [229] made the initial discovery. The Aβ and tau tangles are surrounded by persistent microglial activation, which causes the loss of the homeostatic role of glial cells, developing a proinflammatory trait and exacerbating neurotoxicity. In the case of neuroinflammation, serum and brain specimens from AD patients include inflammatory mediators such TNF-α [230], IL-6, IL-β [231], and cyclooxygenase-2 (COX-2) [232].

## 4. Risk Factors Associated with Alzheimer’s Disease

**Figure 6:**
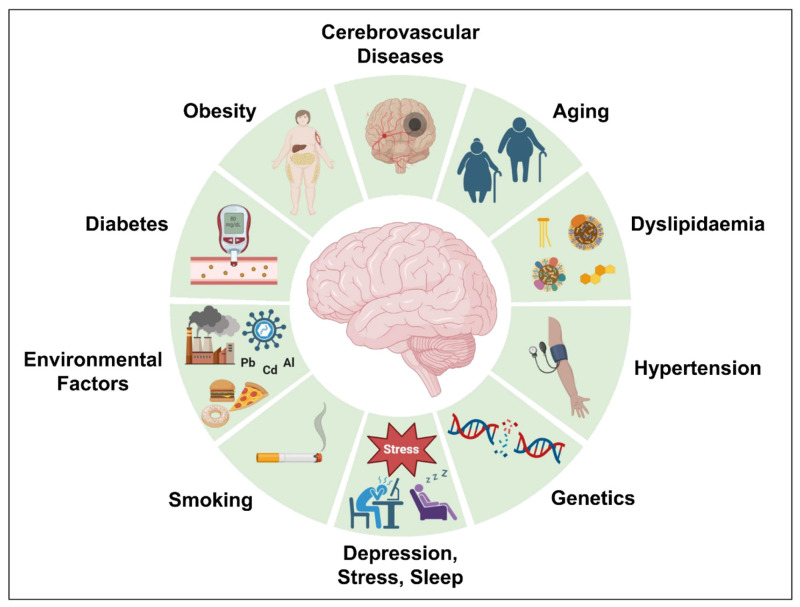
Different risk factors attributed to AD development.

Genetics account for almost 70% of the chance of developing AD. However, acquired variables such as obesity, dyslipidemia, diabetes, hypertension, cerebrovascular disorders, and others raise the possibility of AD development. Several risk factors attributed to AD development [233] including aging, cerebrovascular diseases, obesity, diabetes, hypertension, dyslipidemia, depression, stress, abnormal sleep, and genetics have been discussed further and are illustrated in Figure 6.

### 4.1. Aging

The principal risk factor for AD is aging. Young people rarely suffer from this disease, and the majority of instances of AD begin decades later, after the age of 65 [234]. Aging is a complicated and irreversible operation that influences many different organs and cellular systems, resulting in reduced brain size and weight, synaptic loss, and the swelling of certain ventricles along with SP deposits and NFT [235]. Many conditions such as glucose hypometabolism, dyshomeostasis of cholesterol, malfunctioning mitochondria, depression, and cognitive impairment may also become apparent as people age. These alterations make it difficult to distinguish nascent AD cases from normal aging cases [236,237].

### 4.2. Cerebrovascular Diseases

The risk factors for cerebral vascular diseases and AD are diverse and often co-occur. Cerebral vascular abnormalities such as hemorrhagic infarcts, vasculopathy, and mild to severe ischemic cortical infarcts might enhance the risk of dementia [238,239]. The investigational data by Liu et al. [240] stated that 6 to 47% of those with dementia also have cerebrovascular disease. These findings raise the probability that homeostatic mechanisms play a role in AD and pose the dilemma of whether dementias involving vascular processes are profoundly distinct from those caused by the build-up of Aβ42 and tau proteins or whether both pathological mechanisms have an additive impact on cognitive activity [241]. In accordance with the “double stroke” hypothesis of AD, vascular risk factors (“first stroke”) cause BBB impairment and a decrease in blood flow to the brain, which results in a reduction in the region’s blood supply (oligemia). Through both amyloidogenic and non-amyloidogenic routes, this event causes neural destruction. Primarily, the dysfunction of BBB induces oligemia and the accumulation of neurotoxic compounds, which are linked to the production of numerous focal ischemic infarcts and microinjuries brought on by hypoxia, resulting in neuronal damage. Vascular injury elevates APP expression and processing along the amyloidogenic pathway, raising the Aβ-peptide level.

Additionally, decreased Aβ peptide clearance is brought on by BBB injury. Amyloid deposition in the brain, also known as a “second stroke,” exacerbates neuronal malfunctioning and hastens the progression of neurodegeneration. The Tau protein is hyperphosphorylated due to both ATP build-up and hypoperfusion, which helps generate NFT [242].

### 4.3. Obesity

It is debatable whether obesity is a particular risk factor for AD. Only alcohol consumption has raised the likelihood of cognitive degradation among the several risk variables linked to cognitive deficits in a significant group of patients. Hypercholesterolemia, obesity, and depression were more likely attributed to a low AD risk [243]. The research concluded that a number of the “traditional” risk variables for AD were not linked to the initiation or progression of the disease. On the other hand, a significant association between obesity and the onset of AD has been found, raising the possibility that metabolic alterations linked to obesity harm the neurological system and cause neuronal death through necrosis or apoptosis by changing neural plasticity [244]. A greater midlife body mass index proportionately raises the likelihood of AD, and obesity has been significantly linked to the prevalence of dementia [245]. Additionally, obesity was attributed to various metabolic syndromes affecting the expression of leptin and adiponectin when combined with vascular risk factors [246].

### 4.4. Diabetes

Diabetes has been associated with AD, which is not strange considering that insulin plays a crucial function as a neuromodulator [247]. First, there is a correlation between AD, hyperinsulinemia, and hyperglycemia, which is brought on by insulin resistance and results in SP deposition, particularly in people who express the APOE allele ε4 [248]. Type 2 diabetes and its precursors, excessive obesity, hyperinsulinemia, and dementia, are related. The risk occurs in middle-aged people but not in older people [249]. Severe cortical thinning, increased caspase concentrations, and profound brain atrophy have been observed in transgenic APP/PS1 mice possessing hyperinsulinemia [250]. An enhanced cognitive impairment and Aβ deposition in the histocompatibility complex were observed in studies employing rats with a high-fat diet containing cholesterol [251]. Diabetes-related genes in the sortitin family of vascular protein sorting-10 domain (VpS10) genes are correlated to AD in diabetics, and their malfunction can result in Aβ accumulation and insulin/glucose malfunction [252].

### 4.5. Hypertension

Cumulative research by Skoog et al. [253] demonstrated that having elevated blood pressure might raise the likelihood of developing AD [254]. Hypertension contributes to the risk factors for AD by inducing cerebral edema in chronic instances, thickening of the vessel walls, and constriction of the lumen, all of which impair cerebral blood flow. The findings depict that cerebral ischemia could result in the aggregation of APP and Aβ and boost the expression of presenilin, a protein implicated in the production of Aβ. The BBB may become non-functional due to hypertension, an occurrence linked to the origination of AD [255].

### 4.6. Dyslipidemia

Raised cholesterol concentrations have been highlighted as prospective risk factors for AD development. Previous investigations have revealed that the cholesterol levels of AD patients are 10% higher than in healthy individuals [256]. The BBB is the main reason through which hypercholesterolemia increases AD susceptibility [257]. Various studies have illustrated that high levels of cholesterol circulation could compromise the BBB integrity. Furthermore, other than an enhanced cholinergic neural malfunction, NFT production, neuronal inflammation, cognitive decline, and cerebral micro hemorrhage occurrence consistent with AD, research on animal models shows that hypercholesterolemia is linked to hiked Aβ-peptide accumulation [258,259].

### 4.7. Depression

Depression in early adulthood increases the likelihood of dementia including AD developing later in life [260,261,262]. According to Zverova et al. [263], individuals having AD and indications of depression had a higher chance for cognitive deterioration in the context of cortisol levels. Wu et al. [264] stated that hippocampal shrinkage and Aβ-peptide deposits were identified in certain middle-aged individuals with significant depression, suggesting that the protein metabolism might be changed in depressed patients.

### 4.8. Stress

The emerging proof indicates that chronic stress may increase the likelihood of developing AD and expedite the course of the illness. Significant links exist between susceptibility to stress and elevated degrees of anxiety and dementia incidence [265]. External and environmental stress can cause psychological stress, which then promotes cellular stress, which is then made worse by oxidative destruction and inflammation [266,267,268]. Psychological stress stimulates the hypothalamic–pituitary–adrenocortical (HPA) axis, which causes the release of glucocorticoids into the blood. These substances then pass through the BBB and activate the mineral corticosteroid receptor and glucocorticoid receptor (GR) in the brain [269,270]. Chronic stress results in long-term stimulation of the HPA axis, persistent receptor depletion, and hippocampus neuronal death. According to the glucocorticoid cascading theory, the etiology of AD may be sensitive to HPA axis malfunction [271,272].

### 4.9. Sleep

A study by Proserpio et al. [273] established a bidirectional relation between sleep abnormalities and AD, with sleep disturbances developing before dementia sets in and often becoming worse as dementia progresses. The risk of developing dementia can also increase due to sleep problems. Studies by Shi et al. [274] showed that people with sleep difficulties had a higher risk of getting dementia. Precisely, people who experience insomnia possess a significant risk of getting AD but not vascular dementia or other types of dementia. Similarly, people with sleep-disordered breathing are more likely to experience vascular dementia, AD, and all-cause dementia.

### 4.10. Smoking

By using a variety of pathways, smoking may influence the chance of acquiring AD. It is well-recognized that it can increase the production of free radicals, causing higher oxidative stress and encouraging immune system pro-inflammatory activity, which in turn activates phagocytes and causes further oxidative damage. Moreover, smoking might result in cerebrovascular ailments, boosting the risk of AD [275,276].

### 4.11. Genetics

Over the years, genetic factors have been explored, and it has been determined that they significantly contribute to the onset of AD. Genetic factors are responsible for 70% of AD cases. It has been estimated that genetics comprise around a 70% chance of getting AD. Early AD typically occurs due to mutations in the PSEN1, PSEN2, and APP, while late-form AD is mainly linked to APOE gene polymorphism, particularly the existence of the ε4 allele [277,278]. Approximately 15% of early-onset autosomal dominant AD instances include over 30 dominant mutations in the APP gene. A total of 80% of occurrences of early AD are attributed to mutations in the PSEN1 gene, while only 5% of cases are linked to mutations in the PSEN2 gene (located at 1q31-q42) [279]. Most of the mutations in the APP gene and PSEN1 increase the Aβ42 to Aβ40 ratio, either by the increased expression of Aβ42, decreased expression of Aβ40, or both. The amyloidogenic process is encouraged by this deregulation, which promotes early Aβ accumulation in the brain tissue [237]. As established by Campion et al. [280], it is thought that additional genes complementary to APP, PSEN1, and PSEN2 are implicated in the etiology of early-onset AD.

The APOE gene, found on chromosome 19, encodes the APOE protein, which is engaged in lipid metabolism [281]. The principal risk factor for late-onset AD is the ε4 allele. When ε4 is present, the probability of developing AD rises by three times in heterozygosity and by twelve times in homozygosis. On the other hand, having the ε2 allele of APOE lowers the likelihood of acquiring AD [282,283].

### 4.12. Environmental Risk Factors

Air pollution, diet, metals, infections, and many other environmental risk factors could cause inflammation and oxidative stress, raising the likelihood of developing AD. Hence, it is crucial to comprehend the environmental factors and how AD relates to them [284,285]. Chronic CNS infections are considered risk factors for AD because they could result in the formation of Aβ plaques and NFT. According to Miklossy and Balin’s [286] research, chronic bacterial infections have been linked to AD. Chlamydia pneumonia infection might promote late-onset AD and raise the chance of developing it by activating astrocytes and cytotoxic microglia, interrupting calcium balance, and inducing apoptosis. The investigation by Itzhaki et al. [286] revealed the presence of herpes simplex (HSV-1) viral DNA in patients who carried the ApoE-ε4 allele, suggesting an elevated risk for AD. HSV-1 could multiply in the brain, which can activate the inflammatory process and elevate Aβ accumulation, leading to neuronal damage and AD development [287].

Copper, zinc, and iron are bio-metals that have a physiological role in biological systems. On the other hand, toxicological elements have no biological purpose (e.g., lead and aluminum) [288]. When aluminum builds up in the body, it engages with proteins and provokes misfolding, agglomeration, and phosphorylation of heavily phosphorylated proteins like the tau protein, which triggers the onset of AD. Aluminum is also bound to plasma transferrin and citrate biomolecules, which could facilitate the aluminum transport to the brain [289]. Lead can quickly penetrate the BBB, affecting neuronal development and synaptic plasticity and inflicting significant damage due to enhanced β-secretase expression and Aβ build-up. Given the self-aggregation of tau and the aggregation of Aβ plaques, the water-soluble carcinogenic element cadmium can pass through the BBB and develop neurological disorders like AD [290].

By introducing different contaminants into the atmosphere, air pollution alters the composition of the atmosphere and has recently been shown to be correlated to AD as well as cardiovascular and respiratory problems. There is a connection between oxidative stress, neuroinflammation, and neurodegeneration in people subjected to air pollution. Enhanced Aβ-42 production, aggregation, and diminished cognitive functioning are all possible consequences of air pollution [291,292].

## 5. Conclusions

Rapid developments in cellular biology over the recent decade have been crucial to comprehending the molecular mechanisms behind AD. The molecular pathology of AD is precisely known to be complex, featuring several theories or hypotheses where countless distinctive factors interact. Intriguing research targets studies on distinct molecular mechanisms that lead to hypoxia and oxidative stress including mitochondrial and vascular pathologic conditions. The principal neuropathological indicators of AD that have been evidently explored are Aβ- plaque formation and Tau (tangles) aggregation. The existing literature suggests that we currently understand the various mechanisms responsible for the onset and progression of AD. Thus, AD could be a set of diseases with comparable APP and Tau abnormalities brought on by several mechanisms. Further research is necessary because none of these hypotheses can fully explain all aspects of the disorder on its own. Facets like the actual root cause of AD including aberrant amyloid β generation and the mechanisms by which it affects neurons are still poorly understood. This review contains good insights concerning recent progress in Aβ- and tau-associated molecular mechanisms and glial dysfunction in AD. Furthermore, risk factors associated with AD pathogenesis have been discussed and generalized. In order to establish effective therapeutic approaches for the treatment of AD where existing pharmacological therapy cannot prevent its onset and progression, new findings that aid in understanding the molecular pathogenesis of AD and its correlations are requisite.

## Data Availability

Not applicable.
